# Baby Boomers in Germany: a secondary data analysis of demographics, regional disparities, healthcare utilization, and mortality

**DOI:** 10.1186/s12889-026-27245-z

**Published:** 2026-04-08

**Authors:** Andreas Kuehnl, Volker J. Schmid, Michaela Olm, Wolfgang Weber

**Affiliations:** 1 Department of Industrial Engineering and Healthcare, Ostbayerische Technische Hochschule Amberg-Weiden, Hetzenrichter Weg 15, Weiden, Germany; 2 Department of Statistics, Bayesian Inference and Spatial Statistics Group, Ludwig-Maximilians-Universität München, Munich, Germany; 3 Regierungsrätin im Bayerischen Staatsministerium für Gesundheit, Pflege und Prävention, Munich, Germany

**Keywords:** Baby boomers, Demographics, Urban-rural disparities, Spatial analysis, Healthcare utilization, Mortality patterns

## Abstract

**Background:**

The demographic shift driven by the aging Baby Boomer cohort represents a substantial burden for our healthcare system. The aim of this study is to analyze the basic demographic characteristics, regional disparities, healthcare utilization, and the causes of death of the German Baby Boomer generation.

**Methods:**

The German Baby Boomer generation, defined as those born between 1955 and 1969 was analysed. Information on population statistics, hospital statistics, and causes of death statistics was obtained from the German Federal Statistical Office. Global and local spatial autocorrelation were analysed using global Moran’s I and Getis-Ord-Gi* statistics.

**Results:**

At the end of the Baby Boomers’ birth period, the registered resident population in Germany born between 1955 and 1969 was 18.20 million. Most Baby Boomers live in cities, metropolitan areas or in North Rhine-Westphalia, eastern Germany, and Baden-Württemberg. The relative share of the population clearly shows a statistically significant unequal distribution, specifically an urban-rural divide and an east-west divide: Baby Boomers are particularly well represented in eastern Germany and in rural regions of western Germany. Between 2010 and 2024, the proportion of Baby Boomers among inpatient hospital treatments increased markedly, from 16% to 28% in men and from 15% to 21% in women. In 2024, circulatory, musculoskeletal, and neoplastic diseases were the most common primary diagnoses. Between 1984 and 2024, deaths among Baby Boomers rose substantially from 1.6% to 16% of all deaths. This increase was accompanied by a shift from predominantly external causes of death to neoplasms and cardiovascular diseases, while mortality from infectious diseases, largely driven by HIV in the 1990s, declined markedly by 2024.

**Conclusions:**

Regarding the Baby Boomer cohort, a disproportionately high demand for healthcare services and social resources may be expected. Our findings highlight the need for regionally tailored, gender sensitive and migration aware planning of healthcare resources. These challenges must be carefully addressed in future health policy planning, with particular attention to the urban-rural disparities.

**Supplementary Information:**

The online version contains supplementary material available at 10.1186/s12889-026-27245-z.

## Introduction

Around the transition from the 2020s to the 2030s, the peak of the demographic wave will reach retirement age in Germany [1]. This large generation of Baby Boomers represents an enormous challenge for society, particularly from a social, financial and health policy perspective [2]. With regard to healthcare utilization, a disproportionate increase in the use of inpatient and outpatient facilities is to be expected due to the relative size of the Baby Boomer generation [3–6]. With regard to those in need of care, a recent care report by Germany’s largest statutory health insurer highlights that securing care and support for people in need will be a major societal challenge in the coming decades [7].

To date, there is no globally standardized definition of which birth cohorts count as Baby Boomers. While internationally the definition of the U.S. Census Bureau [8, 9] or the Pew Research Center [10] is often cited (birth years 1946–1964), in Germany the period from 1955 to 1965 or 1969 is more frequently used [11–15]. In addition to the more commonly used 5-year period limits, in one report, the defining criterion for Baby Boomers was set at the size of the German birth cohort of > 1.2 million, which defines the Baby Boomer generation as people born between 1957 and 1968 [1]. The rationale behind these thresholds, whether arbitrary or empirical, remains unclear. However, the smooth increase and decrease in the birth rate in Germany does not provide a clear graphical boundary point for a sharp demarcation of the baby boom. While the birth rate in the USA peaked in 1957 with 4.3 million births [8, 16], this was only reached in Germany a few years later in 1964 with 1.4 million births [1]. This difference may be due to different demographic and social trends as well as the economic development in Germany after the Second World War, e.g. the increase in the number of marriages, reduction in the age of marriage, and a growing willingness to have children [17]. The end of the Baby Boomer years was also delayed in Germany compared to the USA. Among other possible causes such as economic changes, housing shortage, social liberalization, changing values, and the changing role of women, this is often attributed to oral contraception, which was first approved in the USA (Enovid [18], 1960) and later in West Germany (Anovlar [19], 1961), and eastern Germany (“Wunschkindpille” Ovosiston [20], 1965). However, social acceptance and ethical evaluation varied greatly in Germany [19]. Nevertheless, the widespread availability of oral contraceptives at the end of the 1960s might have led to the so-called pill-slump (“Pillenknick”) [21] and thus to the end of the Baby boom.

With increasing age, physical strength and general abilities decline, and the likelihood of illness increases, along with the need for healthcare. In 2025, Baby Boomers will be between 56 and 70 years old, a period in which the incidence and prevalence of common diseases such as cardiovascular disease, neoplasms, type 2 diabetes mellitus, osteoarthritis, and dementia will increase significantly [22]. Profound scientific investigation of the German Baby Boomer cohort is considered essential because their impending transition into advanced ages will disproportionately reshape morbidity profiles and care needs, thereby exerting structurally determinative effects on health-care utilization, workforce capacity, and long-term financing sustainability across the German health system. In order to provide a basis for further health-specific analyses, this routine data analysis is to describe the basic demographic and epidemiological characteristics, regional disparities, the use of healthcare services, and the causes of death of the German Baby Boomer generation.

## Methods

### Data sources

The selection of the analyses carried out was based on the data sources and documentation periods available in Germany. Information on population, hospital, and causes of death statistics was obtained from the GENESIS database of the German Federal Statistical Office. The unique ID numbers of the GENESIS tables are listed in the legends of the corresponding tables and figures. The maximum time periods provided by the Federal Statistical Office were used for the respective statistics. This means that the maximum time period for which data was available from the Federal Statistical Office was always analyzed. The data from the Federal Statistical Office GENESIS database was mainly available in 5-year blocks for data protection reasons. Aggregated 5-year data could therefore only be used every 5 years to ensure accurate classification of Baby Boomers. In principle, the most recent reporting year was always used. In addition, older statistics from before 1990 were taken from the statistical yearbooks of the Federal Republic of Germany (FRG), of the German Democratic Republic (GDR), and of the city of Berlin. The population figures always refer to December 31 of the respective reporting year.

### Inclusion criteria and variables of interest

#### Demographic and migration data

For this study, the definition of Baby Boomers used by the German Federal Institute for Population Research [12] was applied, as this period (1955–1969) was also used in several other German studies [13–15, 23]. Thus, the cohort of interest is the German Baby Boomer generation, i.e. those born between 1955 and 1969. The number of births in the years 1955 to 1969 and the annual migration movements to and from Germany were analyzed. In addition, the annual mortality rate was included in order to plot the cohort size over the course of time.

#### Spatial analysis

The data on the regional demographics of Baby Boomers refer to counties and independent cities (NUTS-3 regions). For reasons of data protection and data availability, all other data refer to the entire federal territory (NUTS-0) and, prior to 1990, to West and East Germany. The regional absolute number of Baby Boomers, their share of the total local population, and the proportion of women among Baby Boomers were analyzed on the basis of districts and independent cities (NUTS-3 regions).

#### Hospital service utilization

The utilization of inpatient hospital services by Baby Boomers was calculated using the so-called “Hospital diagnosis data” from German hospitals using ICD-10 codes. German Diagnosis Related Group (DRG) data are routinely collected, case-based hospital administrative records structured under the G-DRG reimbursement system, providing standardized information on diagnoses, procedures, length of stay, and discharge outcomes for billing and performance monitoring rather than for primary research purposes. The documented main hospital diagnoses and the services provided were analyzed according to the German operation and procedure codes (OPS). Hospital cases were grouped according to ICD-10 chapters I–XXII and the main OPS chapters Diagnostics/Imaging (1, 3), Operations (5), and Medication/Other Measures (6, 8, and 9). In addition, the causes of death in the Baby Boomer cohort were analyzed on the basis of the official causes of death statistics. German statistics on causes of death have long-known weaknesses, which occur both in determining the underlying cause of death and in coding it. These uncertainties must be taken into account when interpreting the data [24–26]. As some of the data was only available in 5-year age groups for data protection reasons, the analysis was carried out at 5-year intervals.

### Statistical analyses

This study is a descriptive study. Categorical variables were presented as absolute numbers and percentages. Moran’s I statistic was calculated to analyze global spatial autocorrelation [27]. The Getis-Ord Gi* statistic was additionally calculated [28]. The Getis-Ord Gi* statistic is a local spatial autocorrelation measure that identifies statistically significant “hot spots” and “cold spots” by comparing the concentration of high or low values around each location to what would be expected under spatial randomness [28]. A significance level of α = 5% was used for all tests. The reporting was based on the STROBE statement and content specific recommendations [29, 30]. Microsoft Excel 2021 and R for Windows 4.5.1 (packages classInt, dplyr, ggplot2, RColorBrewer, sf, spdep, stringr, tidyr, tmap, and writeexl) were used for data processing and graphical presentation.

## Results

### Demographics

**Fig. 1:**
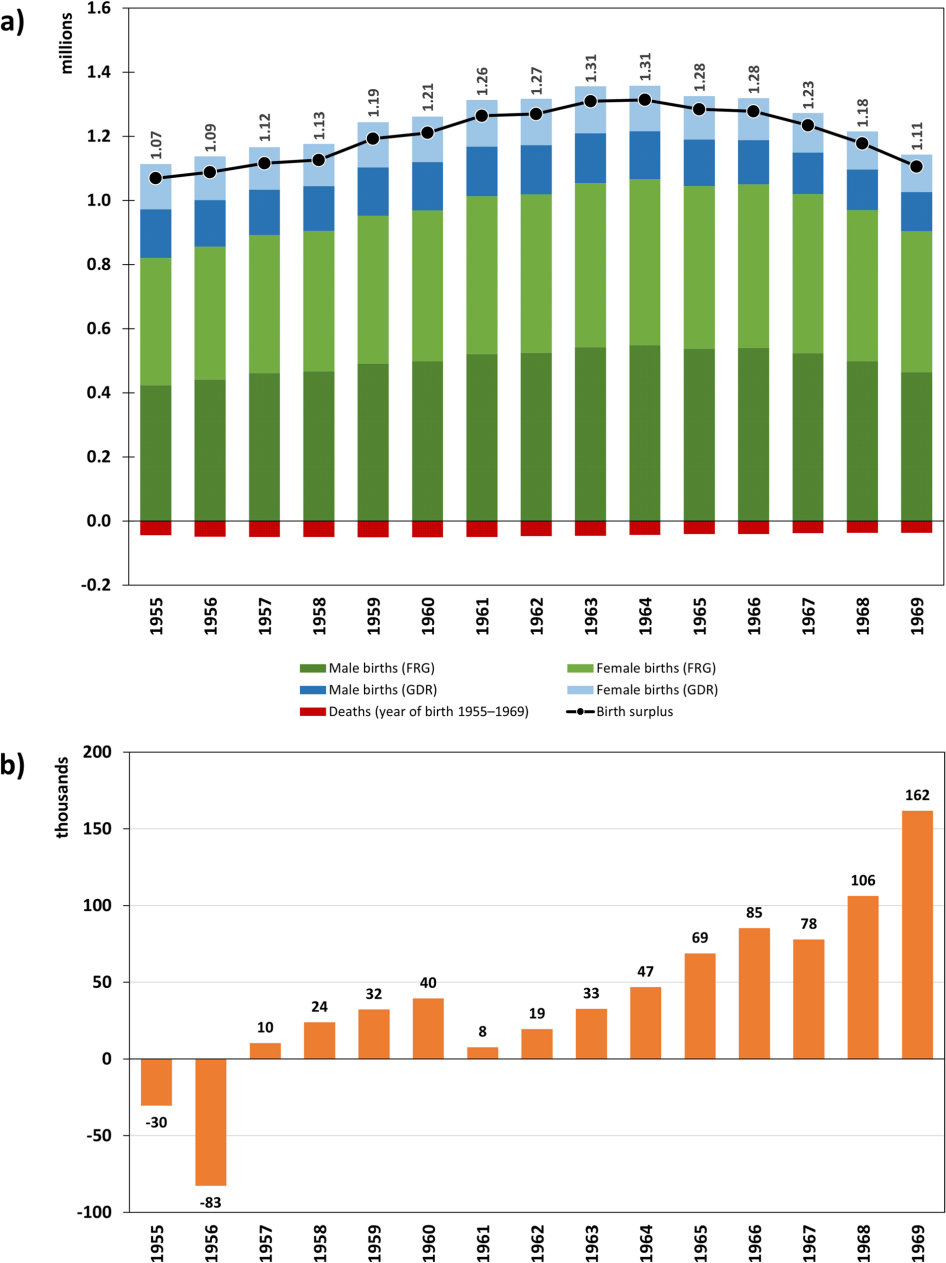
Baby Boomers born in Germany (**a**) and the annual difference between the resident population and the aggregate sum of birth surpluses (corresponds to net migration) for the birth cohorts 1955–1969 (**b**). FRG = Federal Republic of Germany (formerly West Germany), GDR = German Democratic Republic (formerly East Germany). Data source: Statistical Yearbooks of the FRG (1955–1971), Statistical Yearbooks of the GDR (1955–1970), Statistical Yearbooks of Berlin (1957–1960), Statistisches Bundesamt (Destatis), GENESIS-Online Database, Table 12411-0013 und Table 12613-0003, own calculations. Explanation: Figure **b** shows the net migration of Baby Boomers, i.e., how many more Baby Boomers were living in Germany in each year (resident population) than had been born and survived in Germany cumulatively up to that point (cumulative birth surplus). Negative figures indicate a net emigration of Baby Boomers

Between 1955 and 1969, a total of 18.71 million people were born alive in Germany. Annual births amounted to 1.11 million in 1955, rose to a maximum of 1.36 million in 1964, and fell to 1.14 million by 1969 (Fig. 1, top). During the same period, 675,899 people born between 1955 and 1969 died, resulting in a cumulative birth surplus of 18.04 million for the years 1955 to 1969 (see Additional file 1, eTable 1). At the end of the Baby Boomers’ birth period, the registered resident population in Germany born between 1955 and 1969 was 18.20 million, resulting in a positive delta of 161,812 Baby Boomers (Fig. 1, below). The afore-mentioned delta is the net migration of Baby Boomers, i.e., how many more or less Baby Boomers were living in Germany in each year (resident population) than had been born and survived in Germany cumulatively up to that point (cumulative birth surplus). Negative figures indicate a net emigration of Baby Boomers.

**Fig. 2:**
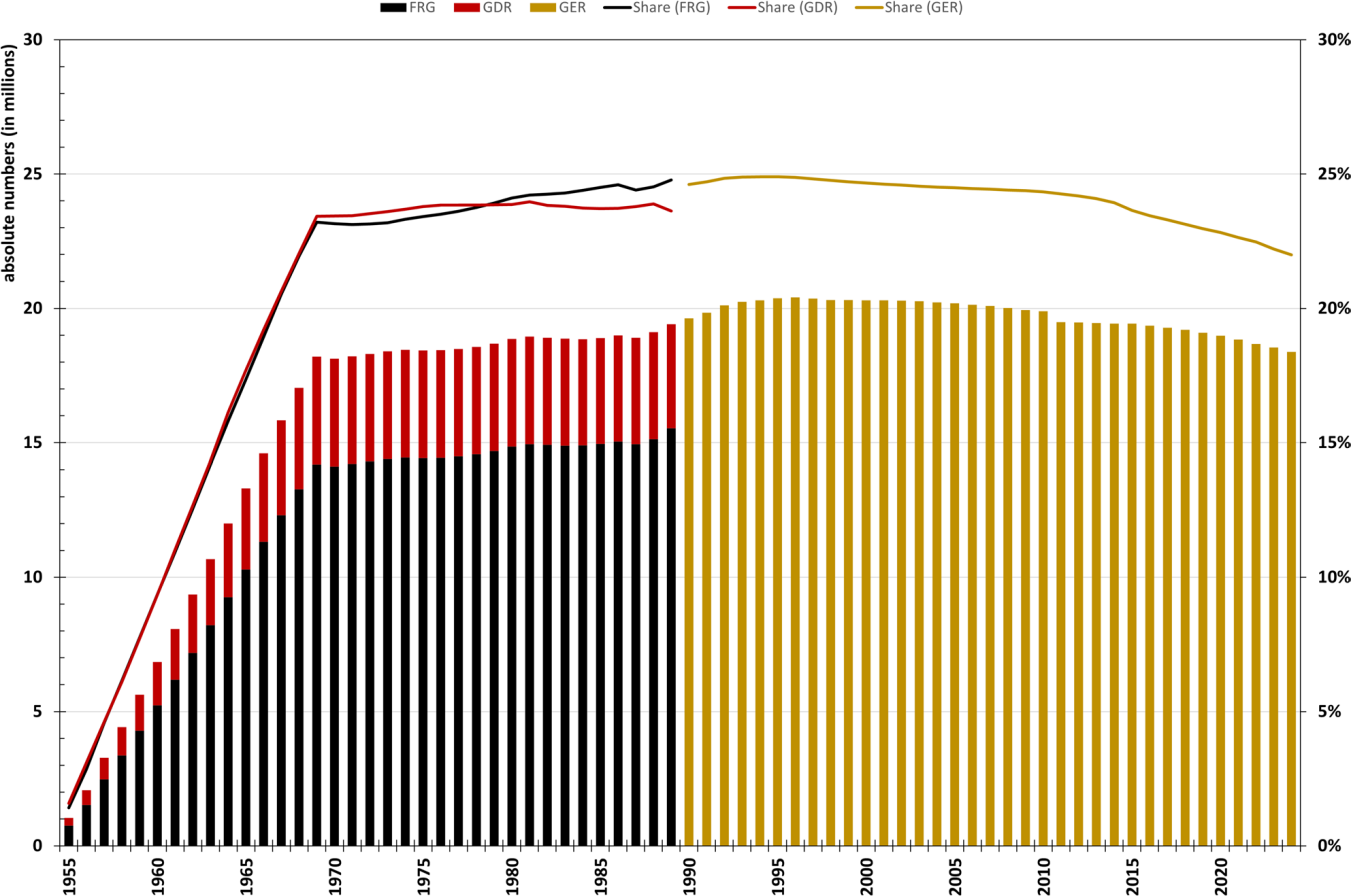
Resident population of Germany born between 1955 and 1969 (absolute numbers, columns) and their share of the total population (lines). FRG = Federal Republic of Germany (formerly West Germany), GDR = German Democratic Republic (former East Germany), GER = Germany. The resident population includes all persons residing in Germany on December 31 of the respective year, regardless of their country of birth. Data source: Statistical Yearbooks of the GDR (1955–1989), Statistical Yearbooks of the FRG (1955–1966), Statistische Jahrbücher Berlins (1957–1960), and Statistische Bundesamt (Destatis), GENESIS-Online Database, Table 12411-0013 (1967–1969) and Table 12411-0005 (1970–2024), own calculations

The total number and proportion of Baby Boomers in the population continued to rise between 1970 and 1989, particularly in West Germany and, after reunification, in Germany as a whole. As more people from the Baby Boomer generation immigrated than emigrated, the original group of 18 Million (birth) became even bigger over time, reaching a peak of 20.40 million (25% of the population) in 1996 (Fig. 2). Overall, these trends indicate a large and persistent Baby Boomer cohort that peaked in the mid 1990s and still accounts for about one quarter of the population.

Age group-specific data on population movements were only available for the reporting years from 2000 onwards and therefore, net migration in earlier decades could not be assessed. This showed that an average of 29,281 German Baby Boomers immigrated each year, while approximately 33,742 German Baby Boomers emigrated (see Additional file 1, eFigure 1). Foreigners born between 1955 and 1969 immigrated at an average rate of 137,058 per year and emigrated at a rate of 118,948 per year. This results in an average net emigration of German Baby Boomers of 4,461 per year and a net immigration of non-German Baby Boomers of 18,110 per year. Data on the nationality of the resident population showed a total of 1.8 million people without German citizenship in the Baby Boomer cohort, corresponding to a foreigner share of 9.9% in 2024 (see Additional file 1, eFigure 2). Regarding family status, in 2024, 65% of Baby Boomers were married, 15% were divorced, 14% were single, and 6% were widowed (see Additional file 1, eFigure 3).

### Regional settlement structure

**Fig. 3:**
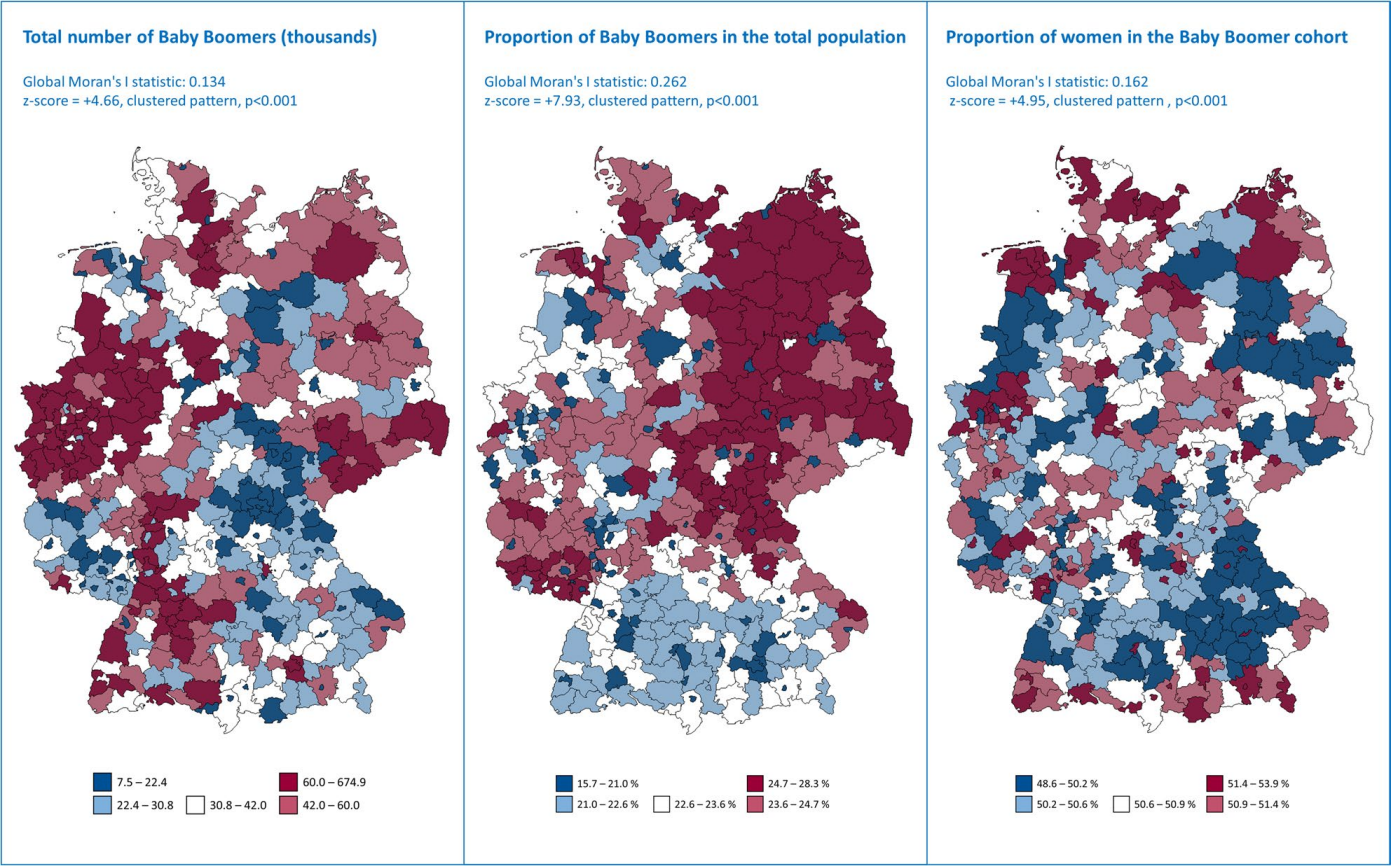
Spatial representation by district and independent city (NUTS Level 3) of the total number of Baby Boomers living in Germany in 2024 (left), the proportion of Baby Boomers in the total population (center), and the proportion of women within the Baby Boomer cohort (right). Data source: Statistisches Bundesamt (Destatis), Regionaldatenbank Deutschland,Table 12411-04-02-4, own calculations

In 2024, among all districts and independent cities, most Baby Boomers lived in Berlin, Hamburg, and Munich, while the fewest lived in Zweibrücken, Suhl, and Ansbach, see Fig. 3 on the left. The relative share of Baby Boomers in the total population ranged from 16 to 28% (median, quartile 1–3: 23, 21–24). The highest proportion of Baby Boomers was recorded in the districts of Spree-Neiße, Altmark Salzwedel, and Uckermark, each with 28% (see Fig. 3 center). The cities of Jena, Leipzig, and Heidelberg had the lowest proportion of Baby Boomers, at 17%, 16%, and 16%, respectively. The proportion of women among Baby Boomers was highest in Cottbus, Coburg, and Schwerin at 54%, 53%, and 53%, respectively. It was lowest in Ludwigshafen am Rhein (49%), Erding (49%), and Offenbach am Main (49%) (see Fig. 3, right). The spatial statistical analysis showed significant hot spots in the number of Baby Boomers in the metropolitan areas of Berlin, Hamburg, and Stuttgart, as well as in eastern North Rhine-Westphalia (see Additional file 1, eFigure 4, left). The proportion of Baby Boomers in the population showed significant hot spots in eastern Germany, Rhineland-Palatinate, and northern Upper Franconia, as well as cold spots in the metropolitan area of Munich, Nuremberg, and Frankfurt (see Additional file 1, eFigure 4, center). The proportion of women in the Baby Boomer population showed isolated hot spots in northern Germany and the Ruhr area. It shows a cold spot in Berlin as well as an extensive cold spot in south Germany (see Additional file 1, eFigure 4, right).

### Use of healthcare services

**Fig. 4:**
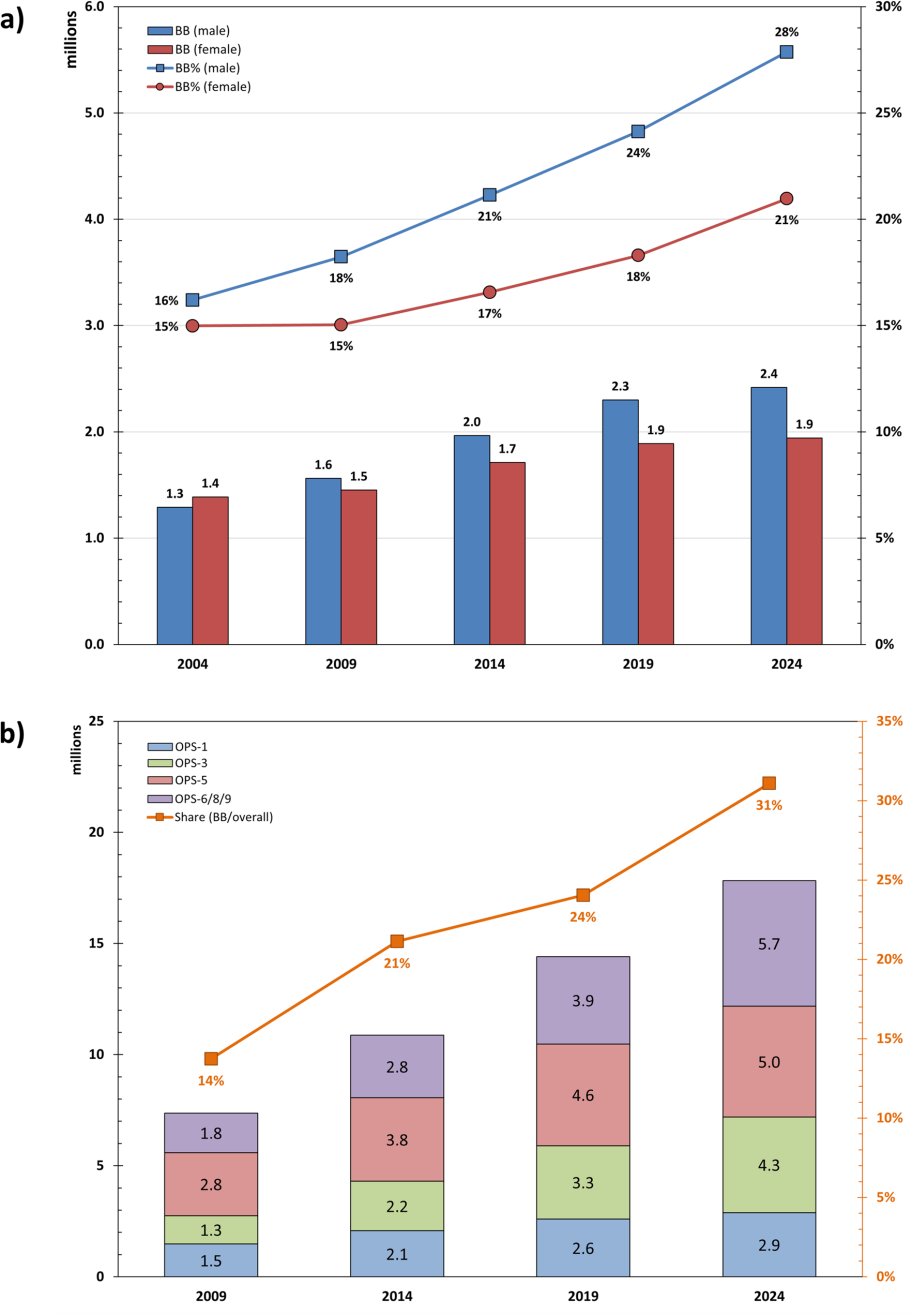
Inpatient hospital cases by sex and reporting year (**a**) and inpatient surgeries and therapeutic procedures (**b**) for those born between 1955 and 1969. The percentages indicate the proportion of Baby Boomers compared to the total population. BB = Baby Boomers, OPS-1 = Diagnostic measures, OPS-3 = Imaging diagnostics, OPS-5 = Surgery, OPS-6/8/9 = Medication, non-surgical therapeutic measures, and complementary measures. Data source: Statistisches Bundesamt (Destatis), GENESIS-Online Database, Table 23131-0002 (**a**) and Table 23141-0102 (**b**), own calculations. Comment: The data sources used for graphs a and b are different. Graph a is based on the so-called "diagnosis data" for hospital patients, which are available from 2004 to 2024. Graph b is based on DRG data, which in Germany has only been available since 2005 (the system was introduced voluntarily in 2003, but complete, uniform, and quality-assured DRG data was only available from the 2005 reporting year onwards)

**Fig. 5:**
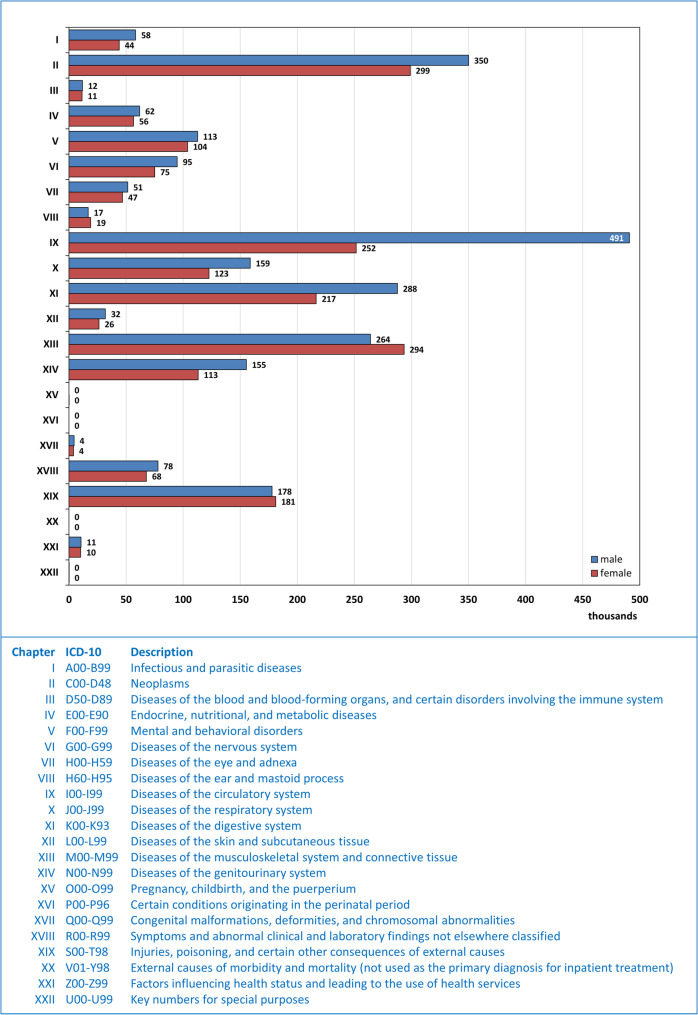
Inpatient hospital cases in 2024 of patients born between 1955 and 1969, by sex and primary hospital diagnosis. Data source: Statistisches Bundesamt (Destatis), GENESIS-Online Database, Table 23131-0002, own calculations

In total, 4.4 million inpatient cases involving patients born between 1955 and 1969 were treated in German hospitals in 2024. Between 2004 and 2024, the relative share of Baby Boomers in all inpatient hospital treatments rose from 16% to 28% for men, and from 15% to 21% for women (Fig. 4a). The most common primary diagnoses in hospitals in 2024 were diseases of the circulatory system (742,657), neoplasms (648,968), and diseases of the musculoskeletal system and connective tissue (557,668). For further details, see Fig. 5.

In 2024, 17.8 million procedures were performed on Baby Boomers in German hospitals, including 2,894,282 diagnostic measures (OPS Chap. 1), 4,302,364 imaging procedures (OPS-3), 4,980,134 surgical procedures (OPS-5), and 5,652,516 drug-based, non-surgical, or complementary measures (Fig. 4b).

### Causes of death

The total number of deaths among Baby Boomers rose from 14,639 in 1984 to 159,702 in 2024, accounting for a rise from 1.6% of all deaths in 1984 to 16% in 2024 (Table 1). In 1984, external causes (e.g., accidents) were the most common cause of death at 67%, followed by neoplasms at 10%, whereas in 2024, neoplasms accounted for 38% of deaths, diseases of the cardiovascular system for 22%, and diseases of the respiratory system for 7.9%. Share of deaths due to infectious diseases peaked in 1994, accounting for 5.7% of all deaths. Of the 1,192 infectious disease deaths, 1,017 (85%) were due to HIV. This proportion fell steadily to 4.2% by 2024. See Table 1 for further details on causes of death. In the present, cardiovascular diseases and neoplasms are responsible for most deaths, reflecting the general mortality patterns in this age group.

**Table 1:** Number of deaths and percentage distribution by year of death and cause of death in the Baby Boomer cohort

Cause of death	1984	1989	1994	1999	2004	2009	2014	2019	2024
Age range of Baby Boomers	15–29	20–34	25–39	30–44	35–49	40–54	45–59	50–64	55–69
Total number of Baby Boomers who died (share of overall deaths per year)	14 639 (1.6)	15 622 (1.7)	20 894 (2.4)	24 754 (2.9)	34 400 (4.2)	49 225 (5.8)	70 941 (8.2)	105 933 (11)	159 702 (16)
Infectious and parasitic diseases	124 (0.8)	404 (2.6)	1 192 (5.7)	630 (2.5)	765 (2.2)	939 (1.9)	1 342 (1.9)	1 457 (1.4)	2 541 (1.6)
Neoplasms	1 495 (10)	2 225 (14)	3 499 (17)	6 031 (24)	10 193 (30)	17 593 (36)	28 348 (40)	42 803 (40)	61 201 (38)
Diseases of the blood and blood-forming organs	60 (0.4)	57 (0.4)	52 (0.2)	51 (0.2)	74 (0.2)	110 (0.2)	150 (0.2)	320 (0.3)	577 (0.4)
Endocrine, nutritional, and metabolic diseases	114 (0.8)	195 (1.2)	310 (1.5)	419 (1.7)	716 (2.1)	1 199 (2.4)	1 964 (2.8)	3 127 (3.0)	5 262 (3.3)
Mental and behavioral disorders	308 (2.1)	915 (5.9)	1 669 (8.0)	1 629 (6.6)	1 665 (4.8)	2 038 (4.1)	2 614 (3.7)	2 976 (2.8)	4 871 (3.1)
Diseases of the nervous system and sensory organs	468 (3.2)	475 (3.0)	476 (2.3)	639 (2.6)	937 (2.7)	1 334 (2.7)	1 975 (2.8)	3 225 (3.0)	5 334 (3.3)
Diseases of the circulatory system	766 (5.2)	1 308 (8.4)	2 442 (12)	4 086 (16)	7 059 (21)	10 129 (21)	14 362 (20)	21 213 (20)	34 665 (22)
Diseases of the respiratory system	367 (2.5)	343 (2.2)	386 (1.8)	514 (2.1)	789 (2.3)	1 575 (3.2)	2 633 (3.7)	5 999 (5.7)	12 670 (7.9)
Diseases of the digestive system	332 (2.3)	750 (4.8)	1 758 (8.4)	2 600 (11)	3 784 (11)	4 719 (9.6)	6 018 (8.5)	8 073 (7.6)	11 013 (6.9)
Diseases of the skin and subcutaneous tissue	10 (0.1)	20 (0.1)	10 (0.0)	9 (0.0)	9 (0.0)	35 (0.1)	86 (0.1)	135 (0.1)	281 (0.2)
Diseases of the musculoskeletal system and connective tissue	24 (0.2)	37 (0.2)	33 (0.2)	49 (0.2)	77 (0.2)	139 (0.3)	206 (0.3)	464 (0.4)	977 (0.6)
Diseases of the genitourinary system	87 (0.6)	87 (0.6)	88 (0.4)	117 (0.5)	155 (0.5)	267 (0.5)	495 (0.7)	918 (0.9)	2 031 (1.3)
Pregnancy, childbirth, and the puerperium	59 (0.4)	43 (0.3)	31 (0.1)	28 (0.1)	12 (0.0)	5 (0.0)	1 (0.0)	0 (0.0)	0 (0.0)
Certain conditions originating in the perinatal period	5 (0.0)	3 (0.0)	4 (0.0)	4 (0.0)	5 (0.0)	12 (0.0)	5 (0.0)	34 (0.0)	76 (0.0)
Congenital malformations and chromosomal abnormalities	206 (1.4)	166 (1.1)	110 (0.5)	95 (0.4)	132 (0.4)	178 (0.4)	314 (0.4)	491 (0.5)	625 (0.4)
Symptoms and abnormal clinical and laboratory findings	438 (3.0)	670 (4.3)	1 113 (5.3)	1 569 (6.3)	1 969 (5.7)	3 219 (6.5)	4 576 (6.5)	8 311 (7.8)	8 806 (5.5)
External causes of morbidity and mortality	9 776 (67)	7 924 (51)	7 721 (37)	6 284 (25)	6 059 (18)	5 734 (12)	5 852 (8.2)	6 387 (6.0)	7 747 (4.9)
Covid-19*	–	–	–	–	–	–	–	–	1 025 (0.6)

Unless otherwise stated, the percentage values refer to the respective column (all deaths within der Baby Boomer cohort)

* = Separate reporting on COVID-19 is available only starting in 2020. Data source: Statistisches Bundesamt (Destatis), GENESIS-Online Database, Table 23211-0002, own calculations

## Discussion

With the Baby Boomer generation, almost a quarter of the German population will leave the workforce and enter retirement age in the next 10–15 years. The transition of the Baby Boomers from working life to retirement poses two challenges for the German healthcare and social system: First, an increasing number of doctors, medical professionals, and social service providers will disappear from the workforce over the next 10–15 years and, second, this increasing number of ageing people will place demands on medical care structures, care facilities, and social insurance structures [31]. Due to the expected long-term changes in population and settlement structure [32], the limited agility in German healthcare reform efforts, as well as the complexity of the healthcare system and bureaucratic hurdles [33], the observed and well-known differences between men and women, and the diverse population from a social and pathophysiological perspective, it is crucial to adopt a forward-thinking, gender-sensitive, and ancestry-aware approach to addressing the healthcare needs of the Baby Boomer generation [31, 34–40].

Due to Germany’s heterogeneous spatial structure, particular attention should be paid to regional aspects relating to Baby Boomers. As Fig. 3 shows, most Baby Boomers live in metropolitan areas or in North Rhine-Westphalia, eastern Germany, and Baden-Württemberg. However, the relative share of the population clearly shows an urban-rural divide and an east-west divide: Baby Boomers are particularly well represented in eastern Germany and in rural regions of western Germany. Their share is significantly lower in cities, which highlights the importance of tailored healthcare planning and future structuring for Baby Boomers, particularly in rural areas.

In 2024, the German Bundesinstitut für Bau-, Stadt- und Raumforschung (BBSR) published a long-term forecast (until 2045) addressing demographic change [32]. The BBSR’s main finding is that the population in Germany is getting older, and that in the framework of the BBSR’s settlement structure district types, peripheral, mostly shrinking districts will have the highest average age in future. At the same time, the institute assumes that regional disparities will continue to increase due to a concentration of population growth in centrally located, structurally stronger districts, mainly in densely populated and metropolitan areas. In terms of regional differentiation, this growth will focus on former West Germany, while population losses will be concentrated in former East Germany (previously the region of the German Democratic Republic, GDR), where many regions already have a high proportion of people of retirement age, partly due to the aftermath of leaving the former GDR by young families after the reunification. BBSR emphasizes the growing challenges for social systems as a result of the increase in the number of people aged 67 and over, a population who belongs to the Baby Boomer generation.

The spatial planning objective of ensuring equivalent living conditions across all regions of Germany, as stipulated in § 2 of the Federal Spatial Planning Act (ROG [41]), is closely linked to demographic change and poses particular challenges for maintaining equitable access to medical and long-term care services, especially in rural, peripheral, and structurally disadvantaged areas. Overall, BBSR assumes that structurally stronger regions can expect a comperatively more positive demographic development (e.g. slower population decline and ageing and more stable or higher levels of immigration and births) to continue until 2045. In contrast, the BBSR predicts population losses and a high intensity of demographic aging in peripheral, structurally weaker and mostly rural regions. This is consistent with our descriptive analyses of the regional distribution of Baby Boomers. In essence, as described by the BBSR [32], spatial disparities in the population structure of Germany will increase over the next 20 years, requiring targeted and regionally adapted healthcare strategies for supporting the aging population.

With increasing age, physical strength and general abilities decline, and the likelihood of illness increases, along with the need for healthcare. In 2025, Baby Boomers will be between 56 and 70 years old, a period in which the incidence and prevalence of common diseases such as cardiovascular disease, neoplasms, type 2 diabetes mellitus, osteoarthritis, and dementia will increase significantly [22]. Among Baby Boomers, a total of 4.4 million inpatient “hospital cases” were treated in 2024 and 17.8 million inpatient procedures (therapeutic or diagnostic measures) were performed, including 5.0 million surgical procedures. With regard to the progression visible in Fig. 4, it can be assumed that these figures will continue to rise in the future and, in absolute terms, the demand for medical services will place greater demands on the healthcare system than in previous generations. These aspects should not be ignored in the context of the planned hospital reform, particularly with regard to rural care close to home.

Since 1984, the relative proportion of deaths among Baby Boomers has risen from 1.6% to 16% (Table 1). While external factors (e.g., accidents) were initially the main cause of death, neoplasms and cardiovascular disease dominated in 2024, which is consistent with the known epidemiology of these diseases given the average age at the time of observation. There was a noticeable increase in infectious diseases as a cause of death in 1994. This is largely attributable to HIV, which was recognized as a separate disease in 1981 and appeared for the first time in German statistical reports on deaths in 1989. Increased awareness, prevention campaigns as well as prophylaxis and therapy options led to the observed decline in deaths in Germany in the following years and decades [42].

However, in addition to purely medical problems, other needs, including those in the social sphere, must not be forgotten, including aspects related to ancestry [43–50]. It can be assumed that the physical and psychological morbidity of Baby Boomers will increase significantly in the future and, given the relative size of the cohort, will require relatively more outpatient, inpatient, financial, and social resources than for the smaller post-war generation and previous generations. Immigration over recent decades has expanded the cohort of residents with non-autochthonous German backgrounds, whose distinct health needs, illness experiences, determinants, behaviors, and risk profiles should be considered in future care planning and in identifying vulnerable groups. Given that loneliness, social isolation, and living alone are important risk factors for all-cause and cardiovascular mortality, particularly in older adults, proactive strategies addressing these social conditions are warranted to improve health outcomes in ageing populations.

This should be carefully considered in all future health policy deliberations: In particular, the marked spatial differences within Germany highlight the possible value of detailed, dynamically updated regional needs analyses that differentiate according to rural and urban settlement structure and demographics, with planning, regional management and financing instruments tailored to underserved regions, especially weak developed rural areas and parts of eastern Germany. In addition, the provision of health services should vary depending on the context: Conurbations may benefit from integrated, 24/7 primary care centers to relieve pressure on emergency rooms, while rural and peripheral regions may rely on telemedicine solutions, mobile practices, and patient-oriented mobility solutions to ensure timely access within specified travel time limits.

To reduce persistent east-west and urban-rural disparities, regionally controlled staffing and institutional arrangements such as community medical centers with permanently employed physicians, dedicated training pathways in general medicine/geriatrics, support services for new or continuing medical practices in peripheral regions and monitoring of routine data on accessibility and waiting times in small catchment areas might improve equity while meeting the multimorbidity and coordination needs of the Baby Boomer generation.

### Limitations

The above-mentioned policy implications are merely considerations derived from the descriptive analysis results and are not based on empirical effectiveness analyses.

In addition, this study has methodological limitations, which will be briefly outlined here. For a detailed description, please refer to the eLimitations (see Additional file 1, eLimitations). There is no uniform international definition of the Baby Boomer generation. Therefore, this study applied the definition of the German Federal Institute for Population Research to ensure comparability with aggregated official statistics.

The terms “Baby boom” and “pill slump” may sound dramatic, but they generally reflect gradual demographic shifts rather than abrupt, large-scale population changes and should be interpreted accordingly.

The analyses are based on administrative data from the Federal Statistical Office, which are subject to data protection constraints, limited age-group resolution, and several methodological restrictions, including structural breaks introduced by the 2011 census revision. Measures of healthcare utilization refer to hospital cases and procedures rather than individual patients, and interpretations of mortality data must consider known limitations in cause-of-death coding and, in some cases, the lack of precise birth-year information. Owing to the use of administrative rather than cohort data, individual characteristics could not be examined, highlighting the need for population-based cohort studies such as the German NAKO study to further investigate the Baby Boomer generation.

## Conclusions

The transition of the Baby Boomers from working life to retirement poses two challenges for the German healthcare and social system: Fewer healthcare providers and more healthcare consumers. Regarding the Baby Boomer cohort, a disproportionately high demand for healthcare services and social resources may thus be expected. Due to Germany’s heterogeneous spatial structure, particular attention should be paid to regional aspects of healthcare provision. However, in addition to purely medical problems, other needs, including those in the social sphere, must not be forgotten. These challenges must be carefully addressed in future health policy planning, with particular attention to the urban-rural disparities.

## Supplementary Information

Additional file 1: Additional text: eDiscussion, eLimitations, Supplementary References. eFigures 1–4. eTable 1.

## Data Availability

All data generated or analysed during this study are included in this published article and its supplementary information files.
