# Supplementary material for: Baby Boomers in Germany: a secondary data analysis of demographics, regional disparities, healthcare utilization, and mortality
Source: BMC Public Health. 2026 Apr 8;26:1228. doi: 10.1186/s12889-026-27245-z (PMC13081385; doi:10.1186/s12889-026-27245-z)
Supplement: Supplementary file 1 — Additional file 1: Additional text: eDiscussion, eLimitations, Supplementary References. eFigures 1–4. eTable 1. [file 12889_2026_27245_MOESM1_ESM.docx]

# Additional material:

## Additional text:

### eDiscussion:

It should be noted at the outset that the terms baby "boom" and "pill slump" sound dramatic and perhaps suggest more significant changes than the phenomena actually had on the population. Both booms and slumps are gradual changes rather than sharply defined, massive changes. This should be taken into account when interpreting the terms used in the text.

With regard to the structure and composition of the cohort, it should not be forgotten that immigration in recent decades has led to the growth of a relevant cohort of non-autochthonous German origin, whose particular health needs, previous experience of illness, health determinants, health behavior and disease risk factors must be taken into account for healthcare and the identification of vulnerable groups in the future.

This study shows that, in addition to the approximately 18 million Baby Boomers born in Germany, early immigration up to 1969 led to an increase in the cohort. This could have been caused not only by resettlers from the former Eastern Bloc, but also by the immigration of so‑called “guest worker families” who arrived in Germany on the basis of recruitment agreements signed by the Federal Republic of Germany until the end of the 1960s; e.g., in 1955 with Italy, in 1960 with Spain and Greece, in 1961 with Turkey, and until 1968 with Morocco, Portugal, Tunisia, and Yugoslavia [1]. Even after that, the cohort and the relative share of Baby Boomers continued to grow, especially in West Germany, while it stagnated in the GDR. It was not until the years around German reunification that the Baby Boomer cohort grew again to over 20 million people, probably due to renewed migration movements in the wake of political upheavals in the states of the former USSR, the Balkan war or labor migration from Eastern Europe. After the end of the 1990s, major immigration peaks of Baby Boomers did not occur until 2015 (crises in Syria, Afghanistan, and Iraq) and 2022 (war in Ukraine).

Since the end of the 1990s, the proportion of foreign Baby Boomers has fluctuated around 10%. However, it should be noted that, according to the Federal Statistical Office, foreigners are persons who do not have German citizenship. It can therefore be assumed that this proportion is higher when German Baby Boomers with foreign roots (but with German citizenship) are included, and this must be taken into account in the context of ancestry‑sensitive provision of health care to Baby Boomers. Furthermore, it should be borne in mind that the (social) determinants of health and disease, genetics, as well as exposure to health risks, in the cohort of immigrant Baby Boomers may differ from those of Baby Boomers born locally [2–9]. In addition to fundamental biological and genetic differences, health behaviors, access to and use of medical services, and the occupational environment, including differential exposure to physical and psychological stressors and hazardous substances, should be taken into account.

With regard to family and living circumstances, two-thirds are married, while one-third are single, divorced, or widowed. Unfortunately, it is not possible to draw conclusions about actual living conditions based on this data, but it can be assumed that a larger proportion of people will be living alone, and that this proportion is likely to increase rather than decrease. This aspect is of increasing importance, particularly with regard to socio-psychological aspects, general care, and, in particular, later health care and nursing care at home. Since loneliness, social isolation, and living alone are significant risk factors for overall mortality and cardiovascular mortality [10], especially among older adults, it seems reasonable to proactively address these social factors in order to improve health outcomes in the aging population [11].

### eLimitations:

1. There's no standard definition of the Baby Boomer generation, either internationally or nationally. For this study, we used the definition from the Federal Institute for Population Research [12], because it covers the alternative definitions and lets us use aggregated data from the Federal Statistical Office. However, the different time periods imply that, when comparing studies on Baby Boomers internationally or within Germany, attention must be paid to the age groups included, whereby the periods that overlap significantly only show slight discrepancies at the margins of the birth periods under consideration.
2. The freely available data, mainly from the Federal Statistical Office, is administrative in nature and, for data protection reasons, does not allow for the observation of, for example, migration movements of individuals or age groups, population distribution at the municipal level, or precise household characteristics.
3. For data protection reasons, some of the data is only available in aggregated age groups, which is why many analyses were only possible in 5-year increments.
4. The measurement of healthcare utilization is based on administrative data. No conclusions can be drawn about individual patients. The data therefore relates to hospital cases and not to patients. It should also be noted that the procedures are also administrative data and that there are usually several procedures per patient. The figures therefore relate to procedures and not to patients.
5. The apparent rise in the share married between 2010 and 2011 is a methods effect from re-basing the population register to the 2011 Census, creating a structural break that limits comparability with earlier years. Destatis documents that, after the switch to the census base, only a partial back-calculation was performed—up to 1 January 2011—so pre-2011 figures were not fully revised to the new basis [13]. From 2011 to 2018, marital status in the population stock was recorded in seven categories (including registered life partnership, partner deceased, and partnership dissolved), which changes category levels relative to the earlier and later four-status scheme. For cohort trends of births 1955–1969 in GENESIS (table 12411-0008), we therefore flag the 2010/2011 step as a structural break rather than a behavioral change.
6. German statistics on causes of death have long-known weaknesses, which occur both in determining the primary cause of death and in coding it. These uncertainties must be taken into account when interpreting the data [14–16].
7. The number of deaths in Table 12613-0003 was only given by age at death, so the year of birth cannot be clearly defined.
8. The presentation of causes of death is purely descriptive. The paper is intended to provide a detailed descriptive analysis and description of the German Baby Boomer generation, which will serve as a basis for further studies and provide fundamental information for other researchers. An analytical comparison of causes of death in relation to other generations was not planned and would therefore go beyond the scope of this study.
9. The data on the resident population of the Federal Republic of Germany from 1955 to 1966 was only rounded to the nearest 1,000, and the population of the German Demokratic Republic for the reporting year 1981 was only available as the average population as of June 30, 1981.
10. Unfortunately, it was not possible to include individual characteristics of Baby Boomers, as this is not a prospective cohort study but administrative data. It would therefore be very interesting to analyze the data from population-based analyses, in particular the German NAKO study [17, 18] with a special focus on the Baby Boomer generation.

Supplementary References

1. Bundeszentrale für politische Bildung. Erstes Anwerbeabkommen vor 65 Jahren. Bundeszentrale für politische Bildung. 20.03.2024.

2. Huebner M, Börnigen D, Deckert A, Holle R, Meisinger C, Müller-Nurasyid M, et al. Genetic Variation and Cardiovascular Risk Factors: A Cohort Study on Migrants from the Former Soviet Union and a Native German Population. Int J Environ Res Public Health. 2021;18:6215. doi:10.3390/ijerph18126215.

3. Osei TB, Mank I, Sorgho R, Nayna Schwerdtle P, Hövener C, Fischer F, et al. Aetiological research on the health of migrants living in Germany: a systematic literature review. BMJ Open. 2022;12:e058712. doi:10.1136/bmjopen-2021-058712.

4. Spallek J, Spix C, Zeeb H, Kaatsch P, Razum O. Cancer patterns among children of Turkish descent in Germany: a study at the German Childhood Cancer Registry. BMC Public Health. 2008;8:152. doi:10.1186/1471-2458-8-152.

5. Zeeb H, Razum O, Blettner M, Stegmaier C. Transition in cancer patterns among Turks residing in Germany. Eur J Cancer. 2002;38:705–11. doi:10.1016/s0959-8049(01)00424-5.

6. Reeske A, Spallek J, Razum O. Changes in smoking prevalence among first- and second-generation Turkish migrants in Germany - an analysis of the 2005 Microcensus. Int J Equity Health. 2009;8:26. doi:10.1186/1475-9276-8-26.

7. Razum O, Zeeb H, Gerhardus A. Cardiovascular mortality of Turkish nationals residing in West Germany. Ann Epidemiol. 1998;8:334–41. doi:10.1016/s1047-2797(97)00232-9.

8. Porsch-Oezçueruemez M, Bilgin Y, Wollny M, Gediz A, Arat A, Karatay E, et al. Prevalence of risk factors of coronary heart disease in Turks living in Germany: The Giessen Study. Atherosclerosis. 1999;144:185–98. doi:10.1016/s0021-9150(99)00054-4.

9. Hasselhorn HM, Peter R, Rauch A, Schröder H, Swart E, Bender S, et al. Cohort profile: the lidA Cohort Study-a German Cohort Study on Work, Age, Health and Work Participation. Int J Epidemiol. 2014;43:1736–49. doi:10.1093/ije/dyu021.

10. Nakou A, Dragioti E, Bastas N-S, Zagorianakou N, Kakaidi V, Tsartsalis D, et al. Loneliness, social isolation, and living alone: a comprehensive systematic review, meta-analysis, and meta-regression of mortality risks in older adults. Aging Clin Exp Res. 2025;37:29. doi:10.1007/s40520-024-02925-1.

11. Hoang P, King JA, Moore S, Moore K, Reich K, Sidhu H, et al. Interventions Associated With Reduced Loneliness and Social Isolation in Older Adults: A Systematic Review and Meta-analysis. JAMA Netw Open. 2022;5:e2236676. doi:10.1001/jamanetworkopen.2022.36676.

12. Bundesinstitut für Bevölkerungsforschung. Aktuelle Meldungen – Bereiche „Bevölkerungsentwicklung“, „Migration“ und „Regionale Unterschiede“ aktualisiert. 01.02.2023. https://www.bib.bund.de/DE/Aktuelles/2021/2021-05-21-Demografische-Fakten-Aktualisierung-Bevoelkerungsentwicklung-Migration-Regionale-Unterschiede.html. Accessed 14 Aug 2025.

13. Statistisches Bundesamt. Bevölkerungsfortschreibung auf Grundlage des Zensus 2011 - Fachserie 1 Reihe 1.3 - 2016.

14. Winkler V, Ott JJ, Becher H. Reliability of coding causes of death with ICD-10 in Germany. Int J Public Health. 2010;55:43–8. doi:10.1007/s00038-009-0053-7.

15. Schelhase T, Weber S. Die Todesursachenstatistik in Deutschland. Probleme und Perspektiven. [Mortality statistics in Germany. Problems and perspectives]. Bundesgesundheitsblatt Gesundheitsforschung Gesundheitsschutz. 2007;50:969–76. doi:10.1007/s00103-007-0287-6.

16. Giersiepen K, Greiser E. Verschlüsselung von Todesursachen für Mortalitätsstatistiken--Vergleich von Signierergebnissen in verschiedenen statistischen Amtern der Bundesrepublik Deutschland und West-Berlins. [Coding of cause of death for mortality statistics--a comparison with results of coding by various statistical offices of West Germany and West Berlin]. Offentl Gesundheitswes. 1989;51:40–7.

17. Peters A, Greiser KH, Göttlicher S, Ahrens W, Albrecht M, Bamberg F, et al. Framework and baseline examination of the German National Cohort (NAKO). Eur J Epidemiol. 2022;37:1107–24. doi:10.1007/s10654-022-00890-5.

18. Wiessner C, Keil T, Krist L, Zeeb H, Dragano N, Schmidt B, et al. Personen mit Migrationshintergrund in der NAKO Gesundheitsstudie – soziodemografische Merkmale und Vergleiche mit der autochthonen deutschen Bevölkerung. [Persons with migration background in the German National Cohort (NAKO)-sociodemographic characteristics and comparisons with the German autochthonous population]. Bundesgesundheitsblatt Gesundheitsforschung Gesundheitsschutz. 2020;63:279–89. doi:10.1007/s00103-020-03097-9.

## eFigures:

| eFigure 1: Immigration to (positive values) and emigration from (negative values) Germany of people born between 1955 and 1969, by nationality. |
| --- |
| 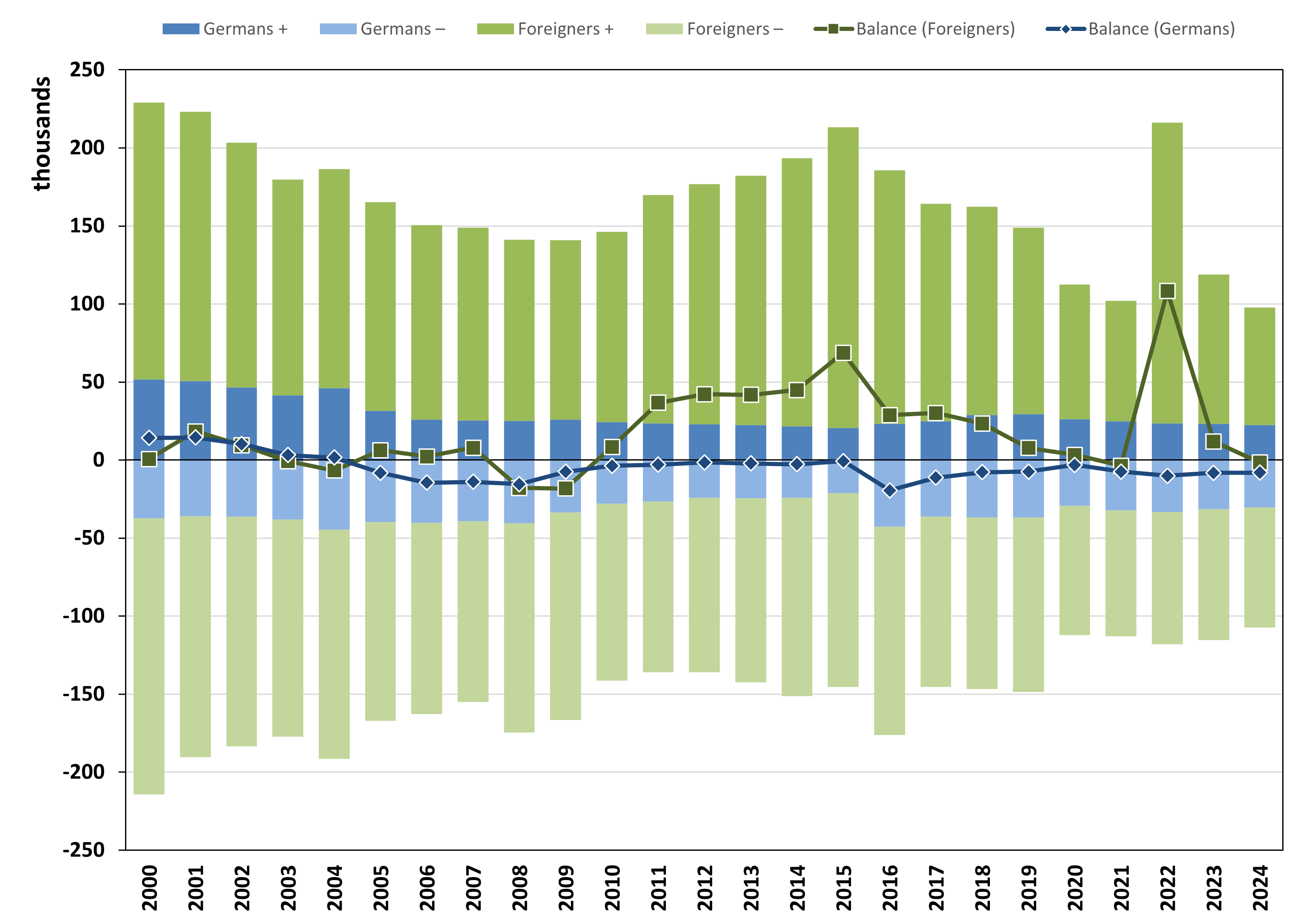 |
| Germans+ = German immigrants, Germans– = German emigrants, Foreigners+ = foreign immigrants, Foreigners– = foreign emigrants. Data source: Statistisches Bundesamt (Destatis), GENESIS-Online Database, Table 12711-0006, own calculations. |

| eFigure 2: Baby Boomers without German citizenship by sex and origin (columns). Total share of the population living in Germany born between 1955 and 1969 (line). |
| --- |
| 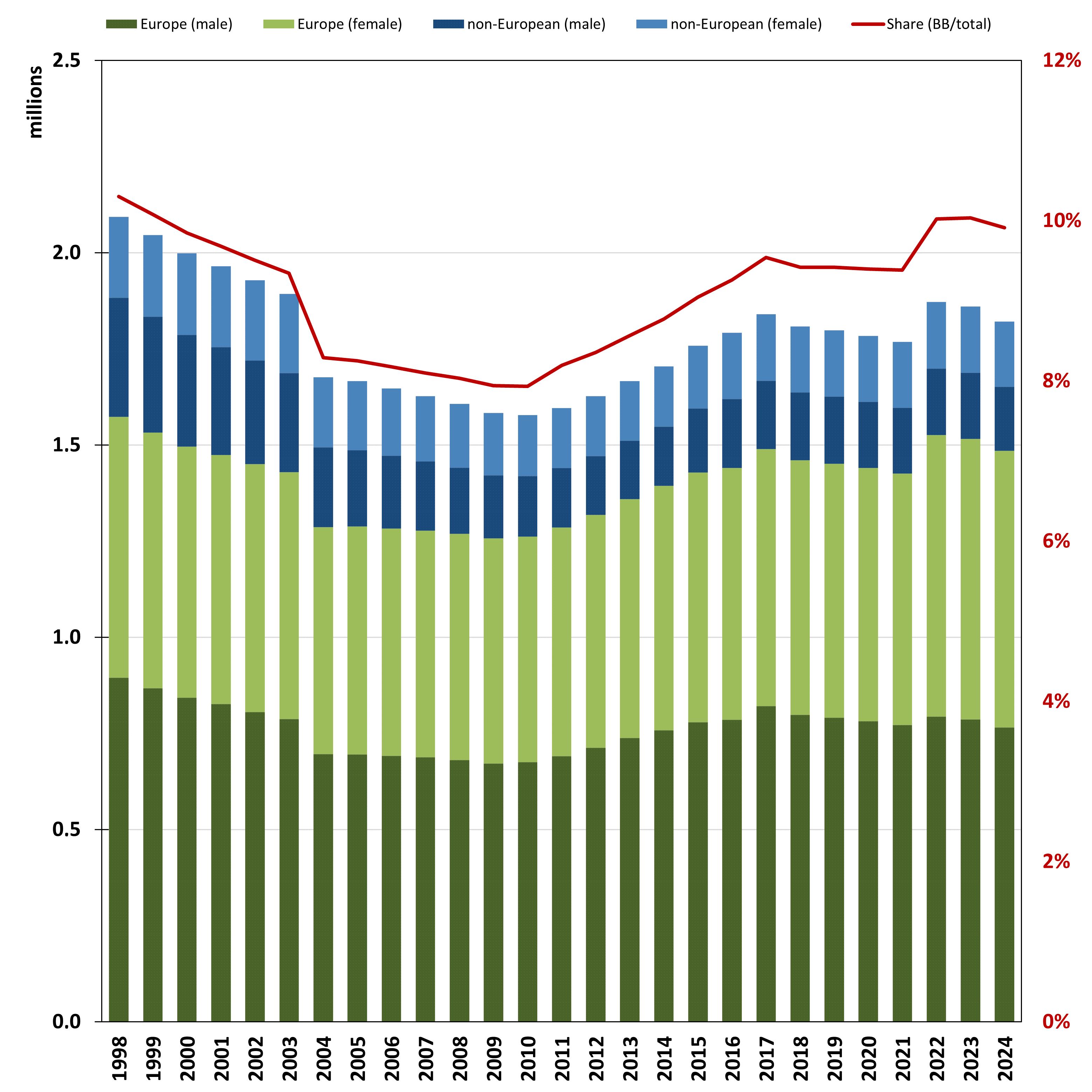 |
| Europe = Originating from other European countries. Non-European = Originating from countries outside Europe. Data source: Statistisches Bundesamt (Destatis), GENESIS-Online Database, Table 12521-0003, own calculations. Note: It should be noted that for the Federal Statistical Office, only German citizenship (passport) is relevant for group allocation, not actual ancestry. |

| eFigure 3: Marital status of people born between 1955 and 1969 (absolute figures on the left, percentages on the right). | |
| --- | --- |
| 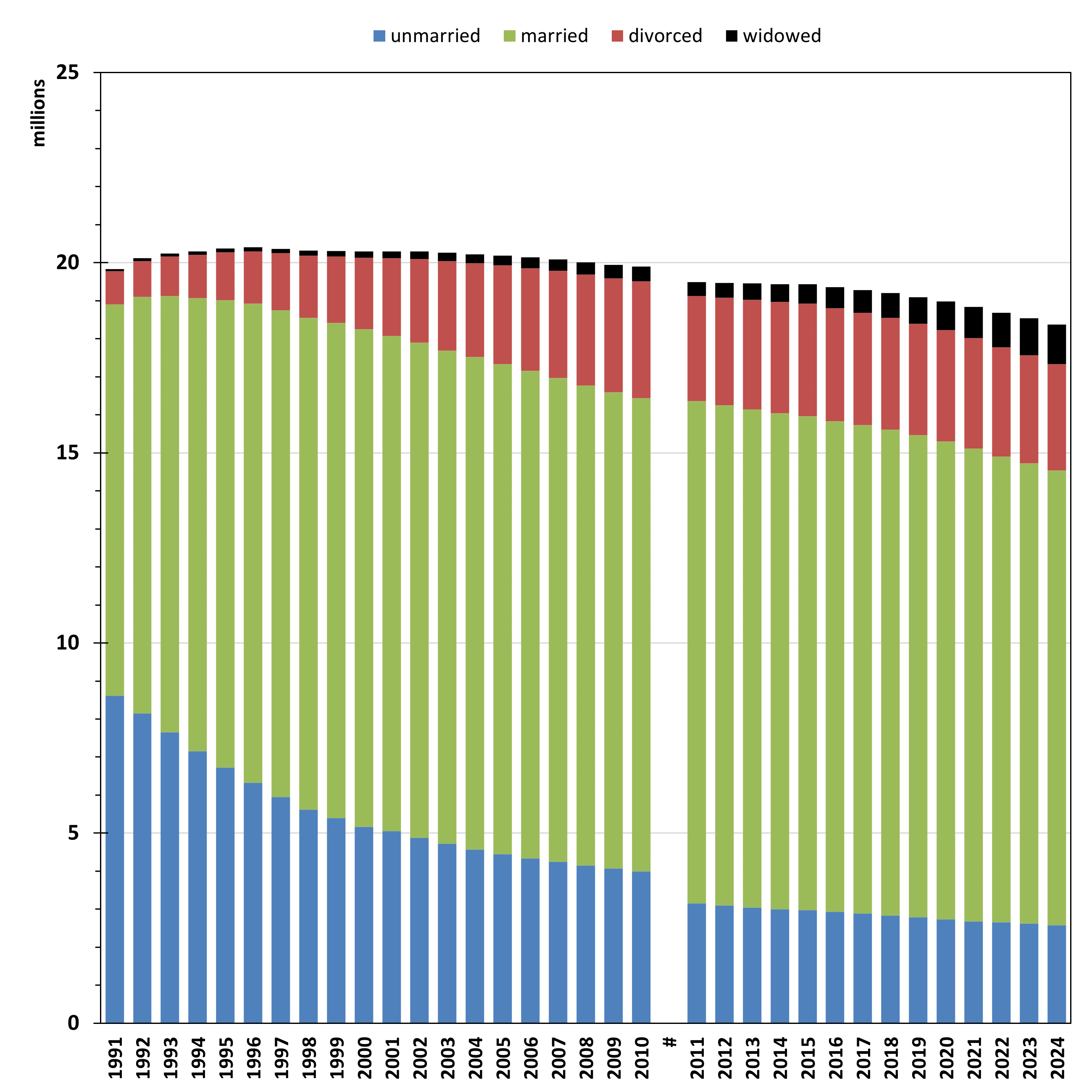 | 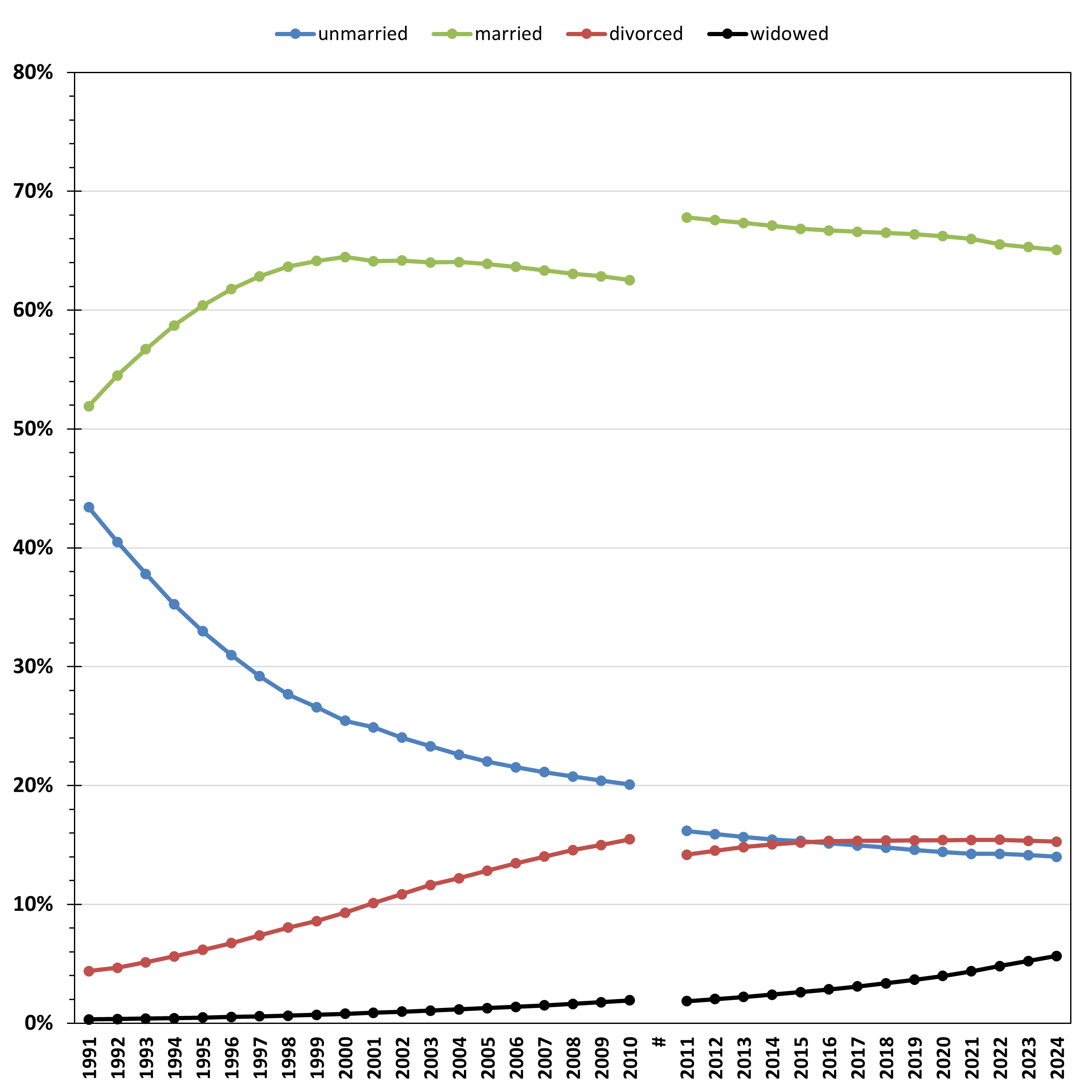 |
| Please note that the break (#) between 2010 and 2011 is purely due to changes in the methods used by the Federal Statistical Office and the 2011 census, and not to changes in the behavior of the population (see limitations). Registered or dissolved civil partnerships and civil partnerships with a deceased partner were added to the categories married, divorced, or widowed, respectively. Data source: Statistisches Bundesamt (Destatis), GENESIS-Online Database, Table 12411-0008, own calculations. | |

| eFigure 4: Local spatial autocorrelation (Hot Spot Analysis) using Getis-Ord G_i_^*^ statistics. Spatial representation is by district and independent city (NUTS Level 3) of the total number of Baby Boomers living in Germany in 2024 (left), the proportion of Baby Boomers in the total population (center), and the proportion of women within the Baby Boomer cohort (right). | | |
| --- | --- | --- |
| Total number of Baby Boomer | Proportion of Baby Boomer in the total population | Proportion of women in the Baby Boomer cohort |
| 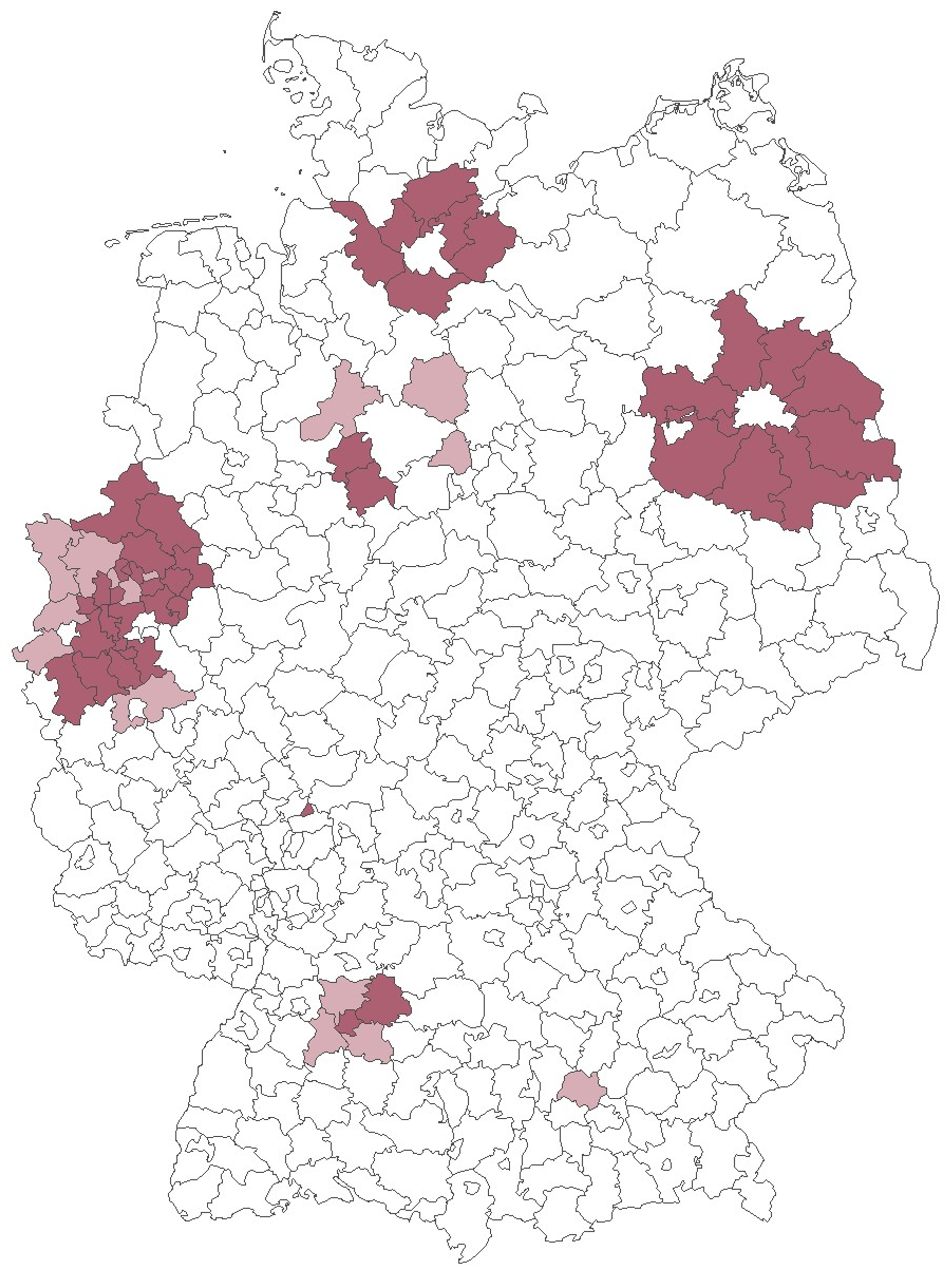  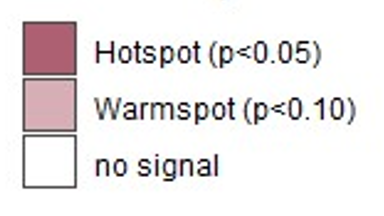 | 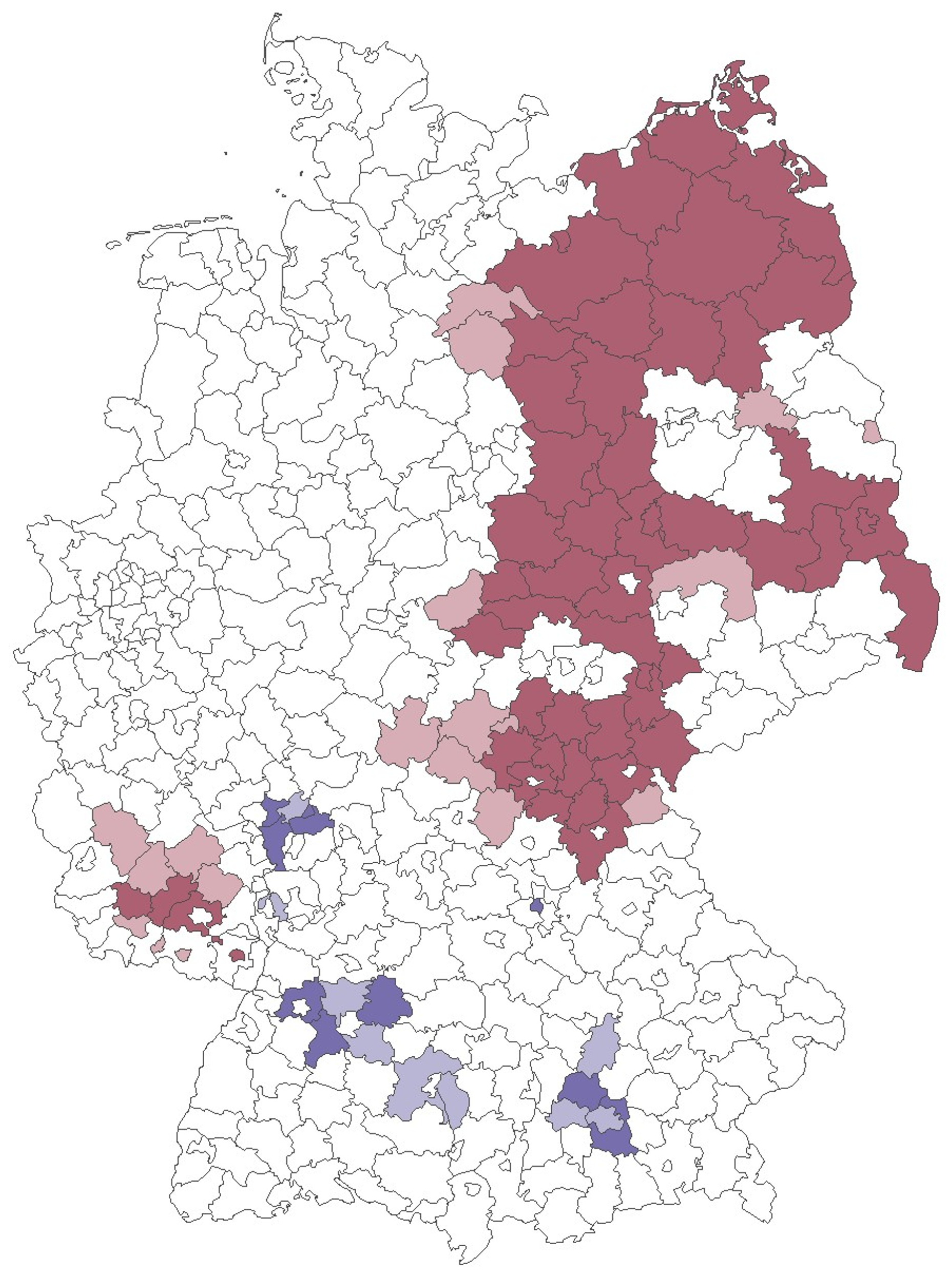  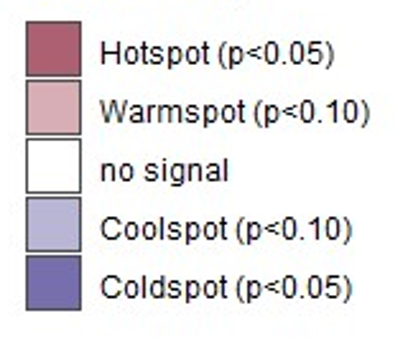 | 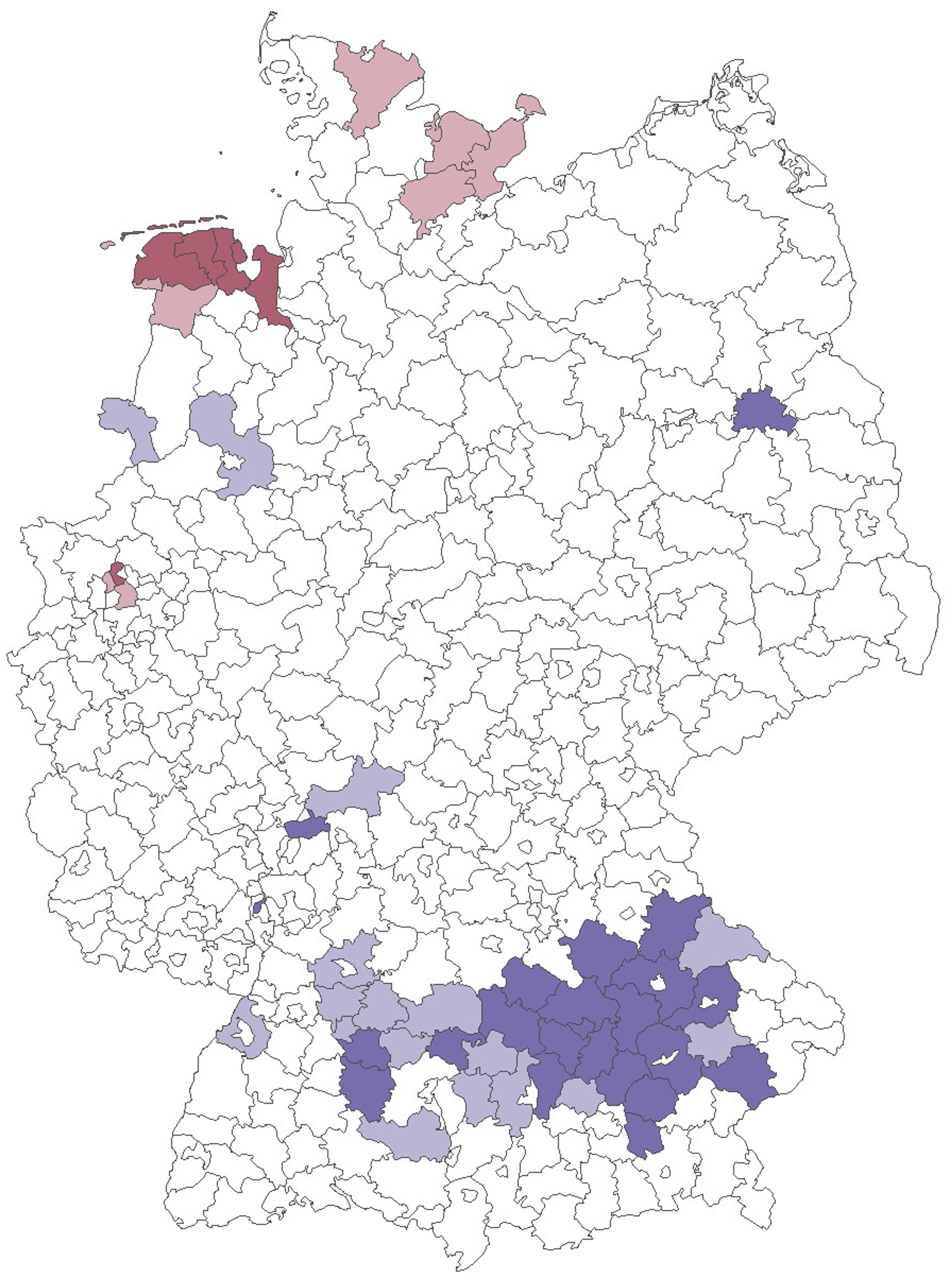  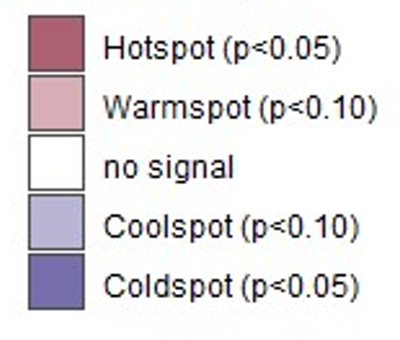 |

## eTables:

| eTable 1: Demographic data of the Baby Boomer generation (births, deaths and resident population). | | | | | | |
| --- | --- | --- | --- | --- | --- | --- |
| **Year*** | **Life births** | **Deaths**^#^ | **Birth surplus**  **(annual)** | **Birth surplus**  **(cumulative)** | **Registered resident population (born 1955-1969)** | **Delta between resident population and birth surplus** |
| **1955** | 1,113,408 | 44,231 | 1,069,177 | 1,069,177 | 1,038,707 | -30,470 |
| **1956** | 1,137,169 | 49,410 | 1,087,759 | 2,156,936 | 2,074,209 | -82,727 |
| **1957** | 1,165,555 | 49,677 | 1,115,878 | 3,272,814 | 3,283,238 | 10,424 |
| **1958** | 1,175,870 | 50,092 | 1,125,778 | 4,398,592 | 4,422,492 | 23,900 |
| **1959** | 1,243,922 | 51,182 | 1,192,740 | 5,591,332 | 5,623,560 | 32,228 |
| **1960** | 1,261,614 | 51,119 | 1,210,495 | 6,801,827 | 6,841,338 | 39,511 |
| **1961** | 1,313,505 | 49,919 | 1,263,586 | 8,065,413 | 8,073,157 | 7,744 |
| **1962** | 1,316,534 | 47,090 | 1,269,444 | 9,334,857 | 9,354,354 | 19,497 |
| **1963** | 1,355,595 | 46,382 | 1,309,213 | 10,644,070 | 10,676,741 | 32,671 |
| **1964** | 1,357,304 | 43,645 | 1,313,659 | 11,957,729 | 12,004,537 | 46,808 |
| **1965** | 1,325,386 | 40,847 | 1,284,539 | 13,242,268 | 13,311,166 | 68,898 |
| **1966** | 1,318,303 | 40,282 | 1,278,021 | 14,520,289 | 14,605,545 | 85,256 |
| **1967** | 1,272,276 | 38,306 | 1,233,970 | 15,754,259 | 15,832,213 | 77,954 |
| **1968** | 1,214,968 | 37,083 | 1,177,885 | 16,932,144 | 17,038,389 | 106,245 |
| **1969** | 1,142,366 | 36,634 | 1,105,732 | **18,037,876** | **18,199,688** | 161,812 |
| * = The data reflect the status as at December 31 of the respective reporting year. All figures refer to the respective valid territorial status of the Federal Republic of Germany and the German Democratic Republic, including the population of Berlin and Saarland.. # = The death figures for 1955 (only for 1955) were not available for the Federal Republic of Germany broken down by year of birth, but only by year of age, which means that children under the age of 1 born in 1954 were probably among those who died in 1955. There is no data available on whether the Baby Boomers who died between 1955 and 1969 were also born in Germany. It is likely that immigrant Baby Boomers were among them. Data source: Statistical Yearbooks of the Federal Republic of Germany (FRG, 1955–1971), Statistical Yearbooks of the German Democratic Republic (GDR, 1955–1970), Statistical Yearbooks of Berlin (1957–1960), Statistisches Bundesamt (Destatis), GENESIS-Online Database, Table 12411-0013 und Table 12613-0003, own calculations. | | | | | | |
